# Persistent alterations of cortical hemodynamic response in asymptomatic concussed patients

**DOI:** 10.2217/cnc-2020-0014

**Published:** 2020-10-28

**Authors:** Allyssa K Memmini, Xin Sun, Xiaosu Hu, Jessica Kim, Noelle K Herzog, Mohammed N Islam, Daniel H Weissman, Alexander J Rogers, Ioulia Kovelman, Steven P Broglio

**Affiliations:** 1 Michigan Concussion Center, University of Michigan, Ann Arbor, MI 48109, USA; 2 Department of Psychology, University of Michigan, Ann Arbor, MI 48109, USA; 3 School of Dentistry, Department of Biologic and Materials Sciences & Prosthodontics, University of Michigan, Ann Arbor, MI 48109, USA; 4 Center for Human Growth and Development, University of Michigan, Ann Arbor, MI 48109, USA; 5 Department of Psychology, University of Toledo, Toledo, OH 43606, USA; 6 Department of Electrical and Computer Engineering, University of Michigan, Ann Arbor, Michigan MI 48109, USA; 7 Department of Emergency Medicine, Michigan Medicine, Ann Arbor, MI 48109, USA

**Keywords:** attention task, mild traumatic brain injury, neuroimaging, reaction time, recovery

## Abstract

**Aim:**

The underlying neurophysiological effects of concussion often result in attenuated cognitive and cortical function. To understand the relation between cognition and brain injury, we investigated the effects of concussion on attentional networks using functional near-infrared spectroscopy (fNIRS).

**Materials & methods:**

Healthy controls and concussed patients, tested within 72 h from injury (T1) and after symptoms resolved (T2) completed a computerized attention task during fNIRS imaging.

**Results:**

T1 patients exhibited slower reaction times and reduced brain activation pattern relative to healthy controls. Interestingly, the cortical oxygenation hemoglobin response at T2 was greater relative to T1 and healthy controls, while reaction time was normative.

**Conclusion:**

The exploratory findings of this study suggest once asymptomatic, a compensatory hemodynamic response may support the restoration of reaction time despite ongoing physiological recovery.

Concussion is a transient, neurological dysfunction resulting from a direct or indirect blow to the head or body causing a rapid head acceleration–deceleration. This leads to alterations of mental status, cognitive function and, in some, loss of consciousness [1]. Concussion is often classified as a functional injury because it is not associated with abnormal structural findings on common neuroimaging techniques, such as standard magnetic resonance imaging or computed tomography scans [1]. Without objective measures, however, appropriate diagnostics and injury management for concussion are clinically challenging.

Following a head injury, as demonstrated in animal models, rapid changes in the neuronal membrane potential causes intense activation of the sodium-potassium pump, necessitating massive amounts of adenosine triphosphate, supported by glucose hypermetabolism [1,2]. Along with other neurochemical responses, the brain moves into a state of ‘energy crisis’ in which cerebral blood flow (CBF) is markedly decreased, further leading to an unbalanced energy supply and demand [2]. Previous work in humans has suggested several areas of decreased CBF following head injury in brain regions commonly associated with the attention network such as the frontal, parietal and temporal regions [3,4]. In addition, this work has revealed decreased task accuracy and slower reaction time when completing attention-based tasks relative to healthy controls [5].

To assess and manage concussions in humans, healthcare providers are encouraged to use a multifaceted approach, including cognitive tasks to measure reaction time, accuracy and working memory. A common sequelae of concussion includes slower reaction time within the first few days of injury with a gradual restoration to baseline values [6]. However, these reaction time measures are likely insensitive to persistent physiological changes once the patient becomes asymptomatic [7] and performs normally on standard clinical assessments.

Along these lines, the current literature has yet to establish a time point at which brain physiology is restored following a concussive event. Despite a growing interest in the principal biologic effect of concussion, few studies have implemented neuroimaging techniques that can appropriately assess CBF post-concussion [4,8,9]. One such technique is functional near-infrared spectroscopy (fNIRS) [9], a cost-effective approach to objectively measure changes in the brain's hemodynamic response, a correlate of neural activity [10], during specific tasks such as neurocognitive assessments.

In the present study, we used fNIRS to assess the hemodynamic response within attention networks of the concussed human brain (1) shortly following injury and (2) after patients were behaviorally asymptomatic. In Experiment 1, we aimed to establish normative behavioral performance and cortical hemodynamic response in healthy adults performing an attentional temporal flanker task during fNIRS neuroimaging. In Experiment 2, we aimed to explore alterations of the hemodynamic response in concussed patients relative to a subgroup of controls from Experiment 1 using the same task. Concussed patients completed the temporal flanker task twice: once during the acute post-injury stage (T1) and again during the asymptomatic recovery stage (T2). Hemodynamic response patterns were compared between the three conditions: concussed T1, concussed T2 and controls. We hypothesized that at T1, concussed individuals would exhibit a reduced cortical hemodynamic response in frontal, parietal and temporal regions during the attention task, relative to controls. We further hypothesized that at T2, concussed individuals would continue to exhibit attenuated cortical hemoglobin responses during the attention task relative to matched controls. Collectively, this project aimed at uncovering neurophysiological alterations following concussion, which may not be completely mitigated after returning to normal symptom thresholds.

## Experiment 1: HbO response during temporal flanker task in healthy adults

### Methods

#### Participants

20 healthy adults (n = 20; 9 males, mean age: 26.30 ± 8.13 years, range: 18 to 51 years) were recruited from Southeastern Michigan, USA in 2018–2019. Participants were enrolled if they did not have a history of neurological or cognitive disorders, were not prescribed medications that may influence cognitive function and had not sustained a concussion within the previous 12 months [11–13].

#### Task description

We implemented a previously described [14] temporal flanker attention task consisting of a large letter at the center of a computer screen, followed by a second, smaller letter. In each trial, participants were asked to identify the second letter as quickly as possible. There were 9 s of fixation at the beginning of each run, with each trial lasting 2 s. Further, each of six 24 s blocks was preceded and followed by 18 s of fixation. Thus, the duration of a task ‘run’ was 279 s (4.65 min). Since there were three runs of the task, the total time was 837 s (13.95 min).

#### Procedure

Prior to enrollment, all participants provided written consent as approved by the university's institutional review board. Upon consent, participants were fitted for the fNIRS cap and completed the temporal flanker task during fNIRS neuroimaging. This study was conducted in accordance of the Declaration of Helsinki.

#### fNIRS neuroimaging & data analysis

**Figure 1.**
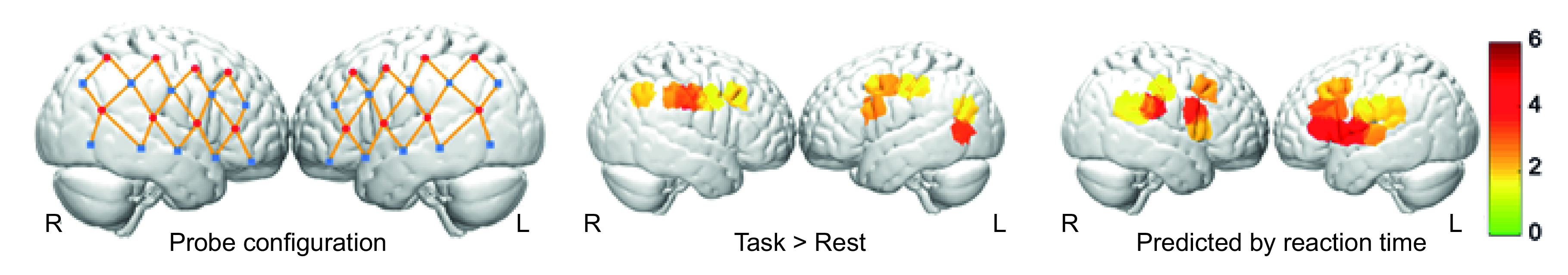
Experiment 1: Functional near-infrared spectroscopy set-up and attention-related brain activity in healthy adults (p < 0.05, uncorrected, color bar reflects t-values).

We used CW6 fNIRS system (TechEn Inc., MA, USA) with 690 and 830 nm wavelengths. The probe configuration consisted of eight light sources and ten light detectors, yielding 24 channels per hemisphere. The data channels covered areas typically engaged in attention processing (frontal, parietal and temporal regions; Figure 1) [3,15]. Data analyses [16] were conducted using NIRS Brain AnalyzIR Toolbox [17], with custom scripts written in MATLAB (MathWorks, MA, USA). Optical density data was converted into HbR/HbO signal for individual data analyses. For the purpose of this study, we focused on the hemoglobin oxygenation (HbO) data as it is a more common index of hemodynamic activity measured with fNIRS, in part, because the 830 nm wavelength that targets HbO is less susceptible to light diffusion than 690 nm [18]. Quantitative analysis indicated that HbR signal changes contributed 16–22%, while HbO signal changes contributed 73–79% to the measured total cortical hemoglobin concentration changes by fNIRS [18]. Individual-level data were analyzed by fitting a general linear model (GLM) with pre-whitening and robust least square solution [19]. A linear mixed effects model was then used to estimate task-related brain activations and brain–behavior relations at the group level using task > resting baseline contrasts. The data were thresholded at p < 0.05 uncorrected, due to the exploratory nature of the study.

### Results

#### Task performance

Across all sessions, participants' accuracy and reaction time were consistent with previously reported findings for healthy adults (accuracy: 97.791 ± 1.733%; reaction time: 0.533 ± 0.103 s) [20].

#### Hemodynamic response

**Figure 2.**
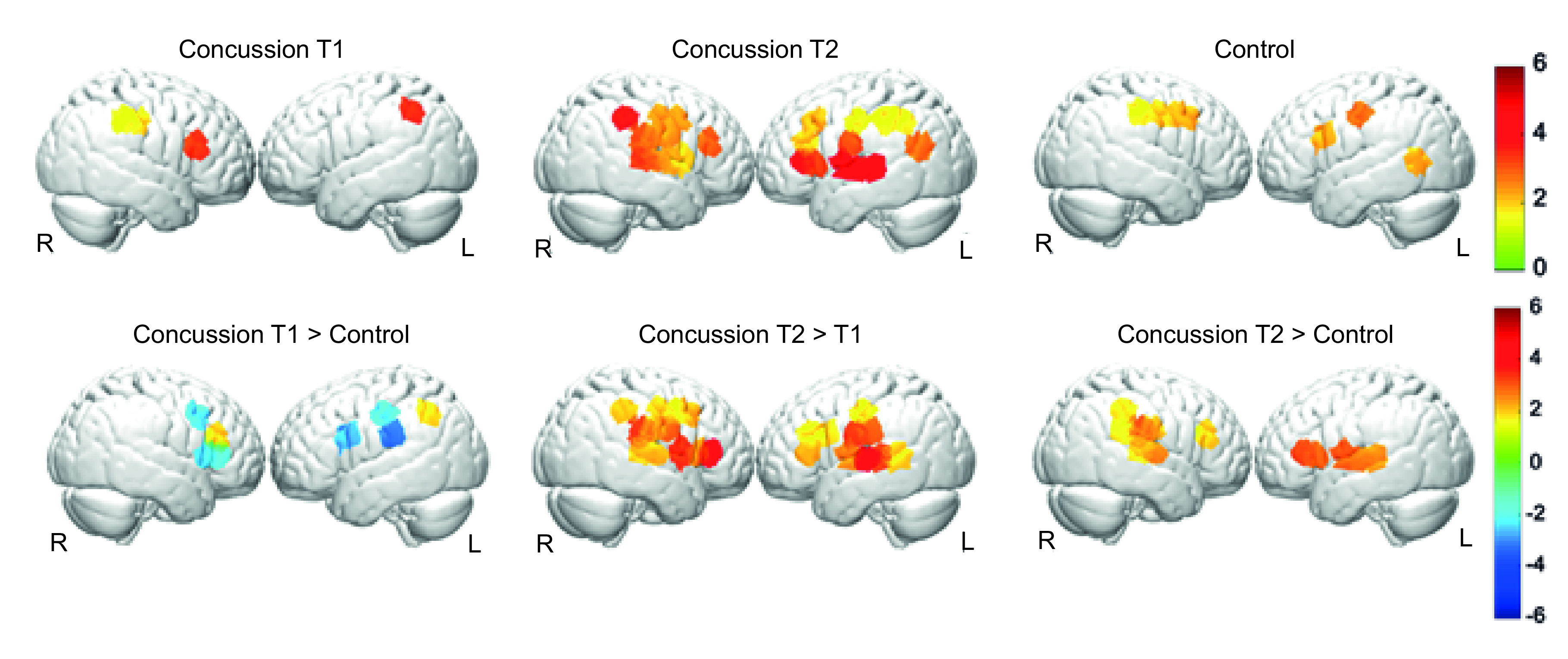
Experiment 2: Attention-related brain activity in concussed individuals (p < 0.05, uncorrected, color bar reflects t-values).

Participants' response patterns for the task relative to rest contrast were consistent with previously reported functional magnetic resonance imaging (fMRI) findings for attention [21]. These response patterns included significant activations in 13 channels, spanning the bilateral prefrontal cortex, bilateral parietal and left occipito-temporal regions (Table 1; Figure 1). Brain-behavior relation analyses revealed that participants with relatively fast overall reaction times showed significantly less activity in 18 channels, including those over bilateral prefrontal, left superior temporal and bilateral parietal regions (Table 1; Figure 2).

**Table 1.** Experiment 1 task versus rest cortical activation all control participants.

	Group/contrast	Regions of interest	β	β-SE	t	p	q	Power (1-β)
		*Left hemisphere*						
Task vs rest	All controls	IFG Pars Opercularis (DLPFC)	7.809	2.802	2.787	0.006	0.060	0.869
		Middle Occipital Gyrus	8.085	2.335	3.462	0.001	0.018	0.963
		Superior Occipital Gyrus	4.657	2.286	2.037	0.044	0.234	0.647
		MFG, DLPFC	4.729	1.934	2.445	0.016	0.128	0.783
		Premotor Cortex	6.480	2.249	2.881	0.005	0.051	0.888
		Primary Motor Cortex	5.677	2.561	2.217	0.029	0.166	0.711
		Primary Sensory Cortex	6.793	2.821	2.408	0.018	0.130	0.772
		*Right Hemisphere*						
		IFG Pars Triangular (DLPFC)	4.220	1.913	2.205	0.029	0.166	0.707
		MFG, DLPFC	4.540	2.264	2.006	0.047	0.239	0.635
		Premotor Cortex	8.057	2.742	2.939	0.004	0.050	0.898
		Primary Motor Cortex	10.089	2.630	3.836	<0.001	0.010	0.984
		Primary Sensory Cortex	6.940	2.371	2.927	0.004	0.050	0.896
		Supramarginal Gyrus	6.246	2.395	2.608	0.010	0.090	0.828
Predicted by RT	All controls	*Left Hemisphere*						
		IFG Pars Opercularis (DLPFC)	45.948	10.618	4.327	<0.001	0.001	0.996
		MFG, DLPFC	34.352	10.067	3.412	0.001	0.012	0.959
		IFG Pars Triangular (DLPFC)	62.741	13.645	4.598	<0.001	0.000	0.998
		IFG Pars Opercularis (DLPFC)	41.772	13.396	3.118	0.002	0.028	0.926
		Anterior STG	55.958	11.232	4.982	<0.001	0.000	0.999
		STG	52.214	11.175	4.672	<0.001	0.000	0.998
		Sensory Motor Cortex	25.755	12.038	2.139	0.035	0.195	0.684
		Posterior STG/MTG	30.959	11.504	2.691	0.008	0.072	0.848
		Supramarginal Gyrus	23.078	11.144	2.071	0.041	0.217	0.660
		Angular Gyrus	29.659	12.013	2.469	0.015	0.101	0.790
		MFG, DLPFC	24.926	9.772	2.551	0.012	0.089	0.813
		MFG, DLPFC	31.069	10.913	2.847	0.005	0.056	0.881
		*Right Hemisphere*						
		IFG Pars Triangular (DLPFC)	31.501	13.911	2.264	0.025	0.153	0.727
		IFG Pars Opercularis (DLPFC)	48.021	12.762	3.763	<0.001	0.004	0.981
		Supramarginal Gyrus	45.810	11.947	3.834	<0.001	0.004	0.984
		Angular Gyrus	23.713	11.868	1.998	0.048	0.223	0.633
		Angular/Superior Occipital Gyri	26.776	13.442	1.992	0.049	0.223	0.630
		MFG, DLPFC	26.463	9.723	2.722	0.008	0.072	0.855
		Primary Motor Cortex	23.793	11.728	2.029	0.045	0.223	0.644

*Note.* Data were analyzed by fitting a general linear model with pre-whitening and robust least square solution. A linear mixed effects model was then used to estimate task-related brain activations and brain–behavior relations at the group level.

DLPFC: Dorsolateral prefrontal cortex; IFG: Inferior frontal gyrus; MFG: Medial frontal gyrus; MTG: Medial temporal gyrus; STG: Superior temporal gyrus.

## Experiment 2: Effect of concussion on HbO response during temporal flanker task

### Methods

#### Concussed participants

Concussed participants were recruited from a local emergency department in Southeastern Michigan, USA, and tested in a laboratory setting within 72 h from initial injury (n = 8; 2 males mean age: 18.38 ± 1.41 years, range: 16 to 21 years, mean concussion history = 0.50 ± 1.07, time from injury = 42.00 ± 16.97 h; symptom severity count = 17.00 ± 2.98, symptom severity score = 60.13 ± 27.81). Participants were enrolled if they were 16–25 years old, presented with a Glasgow Coma Scale greater than 13, a symptom severity score greater than 10 [22], were not prescribed medications that may influence cognitive function, and if they did not have a history of cognitive comorbidities that would influence their performance on the temporal flanker task.

#### Control participants

A subset of eight healthy participants from Experiment 1 was matched to the concussed group on age and gender (n = 8; 2 males, mean age: 20.87 ± 1.86, range: 18 to 23 years).

#### Procedure & data analysis

Prior to enrollment, all participants provided written consent, or assent as appropriate, as required by the university's institutional review board. Participants were then fitted for the fNIRS cap and completed the same neuroimaging task as in Experiment 1. Concussed participants completed the same experimental procedure at Time 1 (T1; symptom severity count = 16.13 ± 4.88, symptom severity score = 49.13 ± 31.89) and at Time 2 (T2; symptom severity count = 2.75 ± 3.20, symptom severity score = 2.88 ± 3.31) visits. After T1, participants' symptoms were remotely monitored using the Sport Concussion Assessment Tool (third edition) symptom survey [23]. Once asymptomatic, concussed participants were scheduled for a follow-up testing session where they completed a second measurement (T2; time between T1 and T2 = 25.50 ± 11.82 days). The control group only completed one testing session (Experiment 1) in order to establish normative data. Paired-samples t-tests were used to identify differences in the behavioral data between T1 and T2. Independent samples t-tests were used to explore differences between the concussed and control groups, with an alpha of 0.05 established *a priori*.

## Results

### Task performance

#### Accuracy

There were no conditional differences in accuracy. First, there were no differences within the concussed group between T1 and T2 (T1: 95.472 ± 4.210%; T2: 97.780 ± 0.961%; t(7) = 2.365; p = 0.189). Second, there were no differences between the concussed group at T1 and controls (controls: 97.001 ± 2.035%; t(7) = 2.145; p = 0.370) or between the concussed group at T2 and controls (t(7) = 2.145; p = 0.345).

#### Reaction time

There was one conditional difference in overall reaction time. Specifically, concussed participants were significantly slower at T1 than at T2 (T1: 0.595 ± 0.139 s; T2: 0.513 ± 0.106 s; t(7) = 2.365; p = 0.010); however, they performed similarly to controls (controls: 0.523 ± 0.087 s; t(7) = 2.145; p = 0.236). At T2, there was no difference between the concussed and control groups (t(7) = 2.145; p = 0.839).

### Hemodynamic response

#### Concussed group: T1 versus T2

At T1, performing the temporal flanker task (vs. resting) was associated with significant activation in four channels within right prefrontal and bilateral parietal regions (Table 2; Figure 2). At T2, the same comparison was associated with significant activation in 23 channels throughout the attention network. A direct comparison between T1 and T2 showed greater activations in 22 channels at T2 compared with T1 (Table 2; Figure 2), covering bilateral frontal, parietal and temporal regions.

**Table 2.** Experiment 2 task versus rest cortical activation in concussed participants and matched controls.

Group/contrast	Regions of interest	β	β-SE	t	p	q	Power (1-β)
	*Left hemisphere*						
T1	Supramarginal/angular gyri	18.369	5.026	3.655	<0.001	0.018	0.976
	Right hemisphere						
	MFG, DLPFC	11.063	3.175	3.485	0.001	0.021	0.965
	Primary sensory cortex	9.604	4.338	2.214	0.029	0.263	0.711
	Supramarginal gyrus	8.805	4.270	2.062	0.041	0.263	0.657
T2	*Left hemisphere*						
	IFG pars opercularis (DLPFC)	12.437	3.118	3.989	<0.001	0.003	0.989
	MFG, DLPFC	6.530	2.782	2.347	0.020	0.098	0.755
	IFG pars triangular (DLPFC)	14.761	4.309	3.425	0.001	0.011	0.960
	STG	15.877	3.468	4.579	<0.001	0.001	0.998
	Sensory motor cortex	12.335	3.629	3.400	0.001	0.011	0.958
	Posterior STG/MTG	15.542	3.933	3.952	<0.001	0.003	0.988
	MTG posterior	16.328	3.996	4.086	<0.001	0.003	0.992
	Superior occipital gyrus	9.204	3.194	2.882	0.005	0.034	0.889
	MFG, DLPFC	6.527	2.622	2.489	0.014	0.075	0.797
	Primary motor cortex	8.759	4.298	2.038	0.043	0.167	0.648
	Supramarginal gyrus	8.157	3.804	2.145	0.034	0.147	0.687
	Supramarginal/angular gyri	8.852	3.885	2.279	0.024	0.111	0.733
	*Right hemisphere*						
	MFG, DLPFC	11.132	3.531	3.153	0.002	0.019	0.932
	Anterior STG	5.780	2.768	2.088	0.039	0.161	0.667
	Primary motor cortex	6.594	3.331	1.980	0.050	0.184	0.627
	STG	9.919	4.113	2.412	0.017	0.087	0.774
	Sensory motor cortex	9.195	3.389	2.713	0.008	0.048	0.854
	Posterior STG/MTG	9.682	3.199	3.026	0.003	0.024	0.914
	Supramarginal gyrus	10.705	4.237	2.527	0.013	0.071	0.807
	MTG posterior	11.817	3.300	3.582	<0.001	0.008	0.972
	Angular gyrus	11.433	3.489	3.277	0.001	0.014	0.946
	Premotor cortex	9.398	3.462	2.715	0.007	0.048	0.854
	Primary motor cortex	9.693	3.632	2.669	0.009	0.051	0.844
	Supramarginal/angular gyri	15.454	3.971	3.891	<0.001	0.003	0.986
Matched controls	*Left hemisphere*						
	IFG pars opercularis (DLPFC)	9.746	3.754	2.596	0.010	0.250	0.826
	Middle occipital gyrus	6.816	2.971	2.294	0.023	0.250	0.738
	Primary motor cortex	11.883	3.869	3.071	0.003	0.247	0.920
	*Right hemisphere*						
	MFG, DLPFC	7.121	2.790	2.553	0.012	0.250	0.814
	Premotor cortex	7.569	2.941	2.573	0.011	0.250	0.820
	Primary motor cortex	8.324	3.477	2.394	0.018	0.250	0.769
	Supramarginal gyrus	7.024	3.295	2.132	0.035	0.306	0.682

Note. Similar to Experiment 1, data were analyzed by fitting a general linear model with pre-whitening and robust least square solution. A linear mixed effects model was then used to estimate task-related brain activations and brain–behavior relations at the group level.

DLPFC: Dorsolateral prefrontal cortex; IFG: Inferior frontal gyrus; MFG: Medial frontal gyrus; MTG: Medial temporal gyrus; STG: Superior temporal gyrus.

#### Concussed group versus control group

Controls showed greater activations in three bilateral frontal and three parietal channels than the concussed group at T1, while their activations were lower in one right frontal and one left parietal channel (Table 3; Figure 2). Contrary to our hypothesis at T2, the concussed group exhibited stronger activations than the control group in 12 channels within bilateral frontal, temporal and right parietal regions (Table 3; Figure 2).

**Table 3.** Experiment 2 cortical activation contrast among concussed and matched-control participants.

Group/contrast	Regions of interest	β	β-SE	t	p	q	Power (1-β)
	*Left hemisphere*						
T1 > matched controls	Supramarginal/angular gyri	13.749	5.866	2.344	0.021	0.197	0.754
	*Right hemisphere*						
	MFG, DLPFC	9.693	4.244	2.284	0.024	0.203	0.734
T2 > T1	*Left hemisphere*						
	IFG pars opercularis (DLPFC)	9.981	4.283	2.330	0.021	0.107	0.749
	MFG, DLPFC	7.838	3.798	2.064	0.041	0.151	0.658
	IFG pars opercularis (DLPFC)	13.454	6.134	2.193	0.030	0.125	0.704
	STG	15.775	5.681	2.777	0.006	0.060	0.868
	Sensory motor cortex	18.178	5.069	3.586	<0.001	0.007	0.972
	Posterior STG/MTG	24.310	4.995	4.867	<0.001	0.000	0.999
	Supramarginal gyrus	14.781	4.719	3.132	0.002	0.024	0.929
	MTG posterior	20.390	5.658	3.603	<0.001	0.007	0.973
	Middle occipital gyrus	14.654	6.464	2.267	0.025	0.120	0.729
	Primary motor cortex	13.208	5.964	2.215	0.028	0.124	0.711
	*Right hemisphere*						
	IFG pars opercularis (DLPFC)	15.396	3.612	4.263	<0.001	0.002	0.995
	IFG pars triangular (DLPFC)	17.895	7.044	2.540	0.012	0.089	0.811
	Anterior STG	12.648	3.503	3.610	<0.001	0.007	0.974
	Sensory motor cortex	15.595	5.016	3.109	0.002	0.024	0.926
	Posterior STG/MTG	11.427	4.808	2.377	0.019	0.101	0.764
	Supramarginal gyrus	11.968	5.515	2.170	0.032	0.127	0.696
	MTG posterior	13.018	5.277	2.467	0.015	0.089	0.791
	Angular Gyrus	16.814	4.655	3.612	<0.001	0.007	0.974
	MFG, DLPFC	12.453	4.969	2.506	0.013	0.089	0.802
	Premotor cortex	10.396	5.229	1.988	0.049	0.173	0.630
	Primary motor cortex	12.563	5.282	2.378	0.019	0.101	0.764
	Supramarginal/angular gyri	14.310	5.605	2.553	0.012	0.089	0.814
T2 > matched controls	*Left hemisphere*						
	IFG pars opercularis (DLPFC)	14.153	4.250	3.330	0.001	0.037	0.952
	IFG pars triangular (DLPFC)	17.511	5.820	3.009	0.003	0.051	0.911
	STG	15.071	4.668	3.229	0.002	0.037	0.941
	Posterior STG/MTG	15.180	5.051	3.005	0.003	0.051	0.910
	MTG posterior	14.043	4.948	2.838	0.005	0.063	0.880
	*Right Hemisphere*						
	MFG, DLPFC	9.763	4.516	2.162	0.032	0.184	0.693
	Posterior STG/MTG	11.952	4.352	2.746	0.007	0.073	0.861
	Supramarginal gyrus	12.359	5.319	2.323	0.022	0.174	0.747
	MTG posterior	10.601	4.773	2.221	0.028	0.184	0.713
	Angular gyrus	15.748	4.813	3.272	0.001	0.037	0.946
	Angular/superior occipital gyri	11.378	5.741	1.982	0.050	0.241	0.627
	Supramarginal/angular gyri	12.110	5.287	2.290	0.024	0.174	0.736

Note. Similar to Experiment 1, data were analyzed by fitting a general linear model with pre-whitening and robust least square solution. A linear mixed effects model was then used to estimate task-related brain activation contrasts and brain–behavior relations at the group level.

DLPFC: Dorsolateral prefrontal cortex; IFG: Inferior frontal gyrus; MFG: Medial frontal gyrus; MTG: Medial temporal gyrus; STG: Superior temporal gyrus.

## Discussion

We compared attentional network activity in healthy adults to that in concussed patients. In Experiment 1, we found that the temporal flanker task engages bilateral prefrontal, bilateral parietal and left occipito-temporal regions in healthy adults. In Experiment 2, shortly after the head impact, concussed participants activated fewer channels and responded more slowly than controls (the former outcome may reflect post-injury vasoconstriction during the acute recovery phase [2]). We also found that after concussed patients became asymptomatic, they exhibited several areas of hyperactivation relative to the controls from Experiment 1 and responded more quickly than they did before. These exploratory findings suggest that after concussed patients become asymptomatic, a compensatory boost of the hemodynamic response supports their ability to respond quickly despite ongoing physiological recovery. This is noteworthy, as current clinical assessments are unable to reliably assess underlying recovery and therefore may put athletes at risk for prolonged recovery or secondary injury if they return to sport prematurely.

Brain–behavior relation analyses involving the healthy controls from Experiment 1 revealed that those who performed the flanker task more quickly exhibited less activity in 18 channels. In other words, as the healthy control participants' task proficiency increased (i.e., faster reaction time), there was less activity observed in the bilateral prefrontal, left superior temporal and bilateral parietal regions, which has also been demonstrated in prior research using an analogous flanker task [24]. In Experiment 2, concussed individuals at T1 demonstrated less brain activity and slower reaction times compared with the controls due to injury-related neurocognitive impairments. These findings are consistent with previous work using fMRI in concussed subjects, indicating areas of low activation of the attention network when assessed with a similar cognitive task [25].

By T2, concussed individuals performed similarly to controls, yet demonstrated activation increases across the attentional network. These results complement the work of Hammeke *et al.*, which suggests injured athletes demonstrated hyperactivation (injured > controls) in the same networks at 7 weeks [25]. Despite exhibiting the same level of behavioral performance as controls, asymptomatic concussed patients may require additional resource allocation (i.e., from the attentional network) in order to perform similarly.

More broadly, the use of neuroimaging in conjunction with clinical behavioral assessments may assist clinicians who wish to determine whether a patient has neurologically recovered following a concussion. At present, many clinicians use subjective symptom inventories to determine whether they should initiate the return to sport protocol [22]. By using non-invasive techniques, such as fNIRS, clinicians may be able to determine whether a patient's hemodynamic responses return to normative levels, which may index a more stable measure of injury recovery. Indeed, unlike fMRI, neuroimaging techniques such as fNIRS provide a cost-effective way to assess the hemodynamic response in patients with a suspected concussion across a wide variety of settings (e.g., an emergency department or sports medicine facility).

Although our findings are novel, we are aware of several limitations. Due to the SARS-CoV-2 outbreak, data collection was terminated prematurely, which led to the concussed and control groups to differ in terms of mean age. Further, concussed participants who sought treatment from the emergency department may have suffered from a more severe concussion than those who typically obtain care from a sports medicine or urgent care clinics (time to recovery for concussed participants = 25.50 ± 11.82 days). The authors also recognize the limited statistical power associated with the exploratory nature and limited sample size of Experiment 2. Lastly, although there is conflicting evidence regarding long-term effects of cognitive deficits after head injury [26], controls were enrolled in the present study only if they did not have a concussion in the previous 12 months. To control for this potential confound, future studies should consider only enrolling control participants without any concussion history. While we regard the study as exploratory given these limitations, the findings are nevertheless supported by previous findings of reduced attentional capabilities and cortical activity in the attention network following concussion [3,4] as well as those suggesting that neural alterations persist after individuals become asymptomatic [25].

In conclusion, the present results suggest pairing fNIRS with a temporal flanker task to assess attentional deficits may yield more nuanced indicators of concussion patients' recovery trajectories than standard behavioral assessments. The use of neuroimaging techniques to assess changes in CBF throughout concussion recovery warrants further investigation as a diagnostic and recovery tool.

## Future perspective

Researchers are eagerly pursuing the development a clinical tool to objectively assess brain structure and function after concussion. Although advanced imaging techniques, such as diffusion tensor imaging and single-photon emission computer tomography, have shown promise, these techniques are costly. Further, they cannot be used to conduct concussion screening on the sidelines, in athletic training facilities or rehabilitation clinics. In contrast, fNIRS is both cost-effective and portable, with recent models featuring wireless data transfer. Therefore, the use of such devices to assess concussion may one day complement current neurocognitive assessments and symptom inventories.

**Executive summary**

- Current concussion assessment protocols rely on clinical functioning and thus may not be sensitive to underlying neural deficits.
- Pairing neuroimaging techniques such as functional near-infrared spectroscopy with clinical behavioral assessments may further assist clinicians when determining if a patient is neurologically recovered.
- The present results indicate that shortly after their injury, concussed patients respond more slowly and exhibit less cortical activation than gender- and age-matched controls.
- In contrast, once concussed patients become asymptomatic, they respond just as quickly as controls and exhibit hyperactivity in bilateral frontal, temporal and right parietal regions.
- These findings suggest that after concussed patients become asymptomatic, a compensatory boost of the hemodynamic response supports their ability to respond quickly, despite ongoing physiological recovery.
